# Protective Role of Polyphenols from Aronia Berry (*Aronia melanocarpa*) Against LPS-Induced Inflammation in Colon Cells and Macrophages

**DOI:** 10.3390/nu17101652

**Published:** 2025-05-12

**Authors:** Shareena Sreedharan, Vimal Nair, Prerna Bhargava, Luis Cisneros-Zevallos

**Affiliations:** 1 Department of Horticultural Sciences, Texas A&M University, College Station, TX 77843-2133, USA; shareenasreedharan@gmail.com (S.S.); vimal.nair16@gmail.com (V.N.); prerna_2kin@yahoo.com (P.B.); 2 Department of Food Science and Technology, Texas A&M University, College Station, TX 77843-2133, USA

**Keywords:** aronia berry, *Aronia melanocarpa*, polyphenols, inflammation, oxidative stress, mode of action, pro-inflammatory genes and nuclear receptor, colon cells, macrophage cells

## Abstract

**Background**: Aronia berry (*Aronia melanocarpa*) are native to North America, rich in polyphenols and antioxidants with the potential to promote human health through its anti-inflammatory properties. **Methods**: Through the chemical characterization of phenolic compounds from aronia berries, 11 distinct polyphenols were identified. We investigated the anti-inflammatory activity of a methanolic/acetone/water extract from freeze-dried aronia berries in LPS-stimulated colonic and macrophage cell models. **Results**: In colon cells, aronia polyphenols suppressed pro-inflammatory gene expression (*NFkβ*, *TNFα*, *IL-6*, *COX2*) by reducing ROS generation while enhancing *LXRα* expression. In macrophages, these compounds decreased NO production through ROS attenuation. Notably, aronia extracts exhibited no cytotoxicity in either cell type across concentrations from 100 to 1000 μg/mL. The whole-berry methanolic extract contained substantial levels of phenolic compounds (including 3-O- and 5-O-caffeoylquinic acids, quercetin derivatives, and cyanidin derivatives) with high ORAC values, likely contributing to their observed multifaceted anti-inflammatory effects. **Conclusions**: These findings suggest that freeze-dried aronia berry (AroBerry^®^) may offer protection against low-grade inflammation, providing a foundation for future in vivo studies using murine models of inflammation-associated chronic diseases to establish appropriate dosage regimens.

## 1. Introduction

Black chokeberry or Aronia berry (*Aronia melanocarpa*) belongs to the Rosaceae family, Maloideae subfamily, and is a deciduous bush tree native to North America. In recent years, interest in aronia berries has increased through its utilization as an ingredient in functional foods [1]. Aronia berries are generally used for preparing jelly, juice, and health products based on their extracts [2,3].

In the early 20th century, the aronia berry was introduced to Russia and Eastern Europe, and since the 1950s it has been commercially grown in that region. Aronia was reintroduced into the U.S. in 1997 in Iowa, with Sawmill Hollow Family Farm being the first aronia berry farm in the United States, owned by the Pittz family. The industry is predominantly located in the Midwest, and at one time, it was estimated that its potential economic impact could be 85 million USD.

Aronia bushes reach maturity at 5 years, with an average production yield of ~10.4 kg per aronia bush in Eastern Europe and ~9 kg per aronia bush in the US. One hectare of aronia bushes could produce around 4000 L of juice after processing. Aronia berries are a good source of vitamin C, with ~13 mg per 100 g of berries [4,5]. Moreover, polyphenols are predominant within aronia berries, and these compounds are involved in its nutraceutical properties [6], in particular its activity against chronic diseases [7]. The total phenolic content in the fruit is ~690 mg per 100 g (GAE) [4], while the anthocyanin content is ~400 mg per 100 g [8]. Moreover, ORAC (Oxygen radical absorbance capacity) analysis reports that aronia berries have a better oxidant scavenging capacity than other traditional fruits, such as blackberries [9]. For these reasons, *Aronia melanocarpa* shows great prospective potential for preventing and treating chronic diseases generally linked with oxidation and inflammation [8,10]. Diabetes and obesity are considered important issues for all healthcare systems due to their economic and social costs. Some clinical tests evaluated *Aronia melanocarpa* and showed the potential of this fruit to help ameliorate these issues. The biological actions of aronia are associated in particular with reductions in total cholesterol amount and improving the lipid peroxidation of red blood cells [11]. Similar action was confirmed from sugar reductions in blood [12]. Additional studies conducted with aronia berries include studies on their antioxidant properties [5], inhibition of cancer cell proliferation and anti-mutagenic effects [13,14,15], hepatoprotective effects [16,17,18], cardio-protective effects, and anti-diabetes effects [12,17,18,19,20,21,22,23]. The prevalence of chronic diseases has been strongly linked to persistent low-grade inflammation, prompting researchers to investigate preventive and therapeutic interventions utilizing dietary supplements, nutraceuticals, and functional foods [24].

Low-grade inflammation can be initiated by diet-induced microbial dysbiosis or obesity, potentially leading to chronic conditions like metabolic syndrome. During inflammatory states, adipose tissue produces harmful adipocytokines including TNF-α (promoting insulin resistance) and PAI-I (associated with thrombosis). This process intensifies as macrophages infiltrate adipose tissue, generating additional inflammatory mediators (TNF-α, MCP-1, and IL-6, NO) in a self-perpetuating cycle that activates inflammatory signaling pathways (JNK and NFκ-β), further exacerbating metabolic dysfunction. These circulating inflammatory factors concurrently affect the vascular endothelium, promoting inflammation that can be amplified by LDL oxidation and infiltration, ultimately contributing to atherosclerosis and platelet-mediated thrombosis [25].

Considering this complex inflammatory network, targeting specific inflammatory components represents a promising approach for preventing and mitigating obesity-related pathologies, including insulin resistance and atherosclerosis.

We propose that aronia berry bioactives exhibit multifunctional anti-inflammatory properties, simultaneously targeting different cellular systems by suppressing oxidative stress and/or modulating nuclear receptor expression, which regulates the pro-inflammatory transcription factor NFκ-β. This study characterizes aronia berry bioactive compounds and elucidates their anti-inflammatory mechanisms in colonic and macrophage cells. Our findings may enhance the value proposition, market potential, and commercial viability of aronia-based products.

## 2. Materials and Methods

### 2.1. Chemicals and Reagents

Lipopolysaccharide (LPS), diphenyleneiodonium (DPI), 2′,7′-dichlorofluorescin diacetate (DCFA), sodium nitrite solution, phenol red-free DMEM/low glucose, Griess reagent, Dulbecco’s Modified Eagle Medium (DMEM)/low glucose, fetal bovine serum (FBS) penicillin/streptomycin mixture, and DMSO were purchased from Sigma (St. Louis, MO, USA). Sodium bicarbonate was purchased from Mallinckrodt Chemicals (Phillipsburg, NJ, USA) and glucose was purchased from Acros Organics (Fair Lawn, NJ, USA). RAW 264.7 macrophages (cell line TIB-71™) and Caco-2 cells were acquired from the American Type Culture Collection (ATCC) (Manassas, VA, USA).

### 2.2. Aronia Berry Samples

Freeze-dried samples of aronia berries (AroBerry^®^) were used in this study and kindly provided by Trim Tab Foods (Sioux City, IA, USA). AroBerry^®^ sources all of their aronia berries from farmers in the United States and is commercially available since 2018. Non-volatile polyphenols were extracted via a methanolic/acetone/water solvent mixture, and the profiles were evaluated using liquid chromatography coupled to mass spectrometry (LC-MS).

### 2.3. Sample Preparation for Polyphenol Analysis and LC-MS Profiling

Lyophilized powder of aronia berry (101.7 mg) was dissolved in a mixture of MeOH/acetone/H_2_O (5:4:1) and stirred for 24 h at 4 °C. The solution was centrifuged at 4000 rpm (2147× *g*) and the supernatant concentrated at 45 °C until all of the volatile solvents were evaporated in a Centrivap concentrator (Labconco, Kansas City, MO, USA).

The phenolic profiles were analyzed using a Surveyor HPLC/MS system with an autosampler, quaternary pump (2000 series), and UV-PDA detector (2000 series) coupled to a C18 reverse phase column (Atlantis, Waters, Ireland; 150 mm × 4.6 mm, 5 μm particle size). The column output was connected to a LCQ Deca XP Max MSn system (Thermo Finnigan, San Jose, CA, USA) equipped with an ESI source and controlled via Xcalibur v1.3 software. Chromatographic separation employed a flow rate of 0.3 mL/min with a mobile phase consisting of water (A) and methanol/acetonitrile (B), both acidified with 0.5% formic acid. The gradient program was as follows: 2/98 to 32/0–34/0, 35/98–40/98 (min/% phase A). Detection was carried out at 280, 320, and 360 nm, with full spectral acquisition from 200 to 600 nm. ESI was performed in the negative ionization mode using nitrogen as the sheath gas (60 arbitrary units) and helium as the dampening gas. The operating parameters included the following: capillary voltage, 45.7 V; spray voltage, 1.5 kV; capillary temperature, 285 °C; tube lens voltage, 30 V; and collision energy, 30% for MS^n^ analysis. The samples were analyzed in triplicate at a 5 mg/mL concentration. Individual phenolics were quantified by cyanidin, quercetin and chlorogenic acid standards in mg equivalents/100 g freeze-dried sample. Total phenolics by the Folin assay and expressed as chlorogenic acid equivalents in mg/100 g freeze-dried sample, while antioxidant activity by the ORAC assay and expressed as mg Trolox equivalent/100 g freeze-dried sample.

### 2.4. Cell Culture for Inflammation Studies

Caco-2 cells (human colon adenocarcinoma, passages 5–20) were maintained in high-glucose DMEM (pH 7.2–7.4) containing 4 g/L glucose, 3.7 g/L sodium bicarbonate, 20% FBS, and antibiotics (100 units/mL penicillin, 100 μg/mL streptomycin) at 37 °C in a humidified 5% CO_2_ atmosphere. For assays including MTS, ROS, and NO, cells were plated at 8 × 10^3^ cells/well in 96-well plates (Costar, Cambridge, MA, USA), while gene expression studies used 0.5 × 10^6^ cells/well in 6-well plates (BD Biosciences, Franklin, NJ, USA). The experimental protocol involved a 5 h aronia extract pretreatment followed by 19 h co-incubation with 50 µg/mL LPS (24 h total exposure to extract).

Raw 264.7 macrophages were cultured in low-glucose DMEM (pH 7.2–7.4) supplemented with 4 g/L glucose, 3.7 g/L sodium bicarbonate, 10% FBS, and antibiotics (100 units/mL penicillin and 100 μg/mL streptomycin) at 37 °C in a humidified 5% CO_2_ atmosphere. Experiments utilized cells between passages 3–11 seeded at 0.5 × 10^4^ cells/well in 96-well black- or clear-bottom plates for viability (MTS test) and inflammatory marker assays (NO/ROS). The experimental protocol consisted of a 5 h aronia extract pretreatment followed by 19 h co-incubation with LPS (1 µg/mL). All extracts were prepared in growth medium containing 0.5% DMSO, with equivalent DMSO-containing media serving as the control.

### 2.5. Cell Viability Test

The extract cytotoxicity was evaluated using the MTS assay (Promega Corp., Madison, WI, USA) following the manufacturer’s protocol. Raw264.7 cells (0.5 × 10^4^/well) and Caco-2 cells (8 × 10^3^/well) were seeded in 96-well plates and incubated for 24 h in DMEM at 37 °C with 5% CO_2_. Cells were then exposed to varying extract concentrations (100–1000 µg/mL) for 24 h. After adding 20 µL MTS solution and incubating for 3 h, the cell viability was determined by measuring formazan production at 490 nm, with absorbance directly proportional to living cell numbers.

### 2.6. Detection of Extracellular Nitric Oxide and Intracellular Reactive Oxygen Species Production

For cellular assays, Raw 264.7 (0.5 × 10^4^/well) and Caco-2 cells (8 × 10^3^/well) were seeded in appropriate 96-well plates overnight before sequential treatment with extracts (5 h pretreatment) and LPS (1 µg/mL for Raw 264.7; 50 µg/mL for Caco-2) for an additional 19 h, with continued extract exposure.

Nitrite accumulation was quantified using Griess reagent. Cell supernatants were collected after the 24 h treatment period and mixed 1:1 with Griess reagent alongside sodium nitrite standards (10–100 µM). Absorbance was measured at 540 nm using a Synergy HT microplate reader (Bio-Tek Instruments Inc., Winooski, VT, USA).

Intracellular ROS generation was determined using DCFA. Following the 24 h treatment protocol, cells were incubated with 10 µM DCFA in phenol red/FBS-free DMEM for 30 min, washed twice, and fluorescence was immediately measured (excitation: 485 nm; emission: 528 nm) using the same microplate reader.

### 2.7. Total RNA and Gene Expression Analysis (Real-Time qRT-PCR)

Following 19 h LPS exposure, RNA was isolated from Caco-2 cells using TRIzol^®^ Reagent (Invitrogen, Carlsbad, CA, USA) and quantified via a NanoDrop ND-1000 spectrophotometer. DNase I-treated RNA (1 µg) was reverse-transcribed using SuperScript III First-Strand Synthesis SuperMix (Invitrogen, Carlsbad, CA, USA). Real-time qRT-PCR was performed with Power SYBR Green PCR Master Mix on a 7900 HT Sequence Detection System (Applied Biosystems, Foster City, CA, USA). Gene expression was normalized to β-actin [18,19] and analyzed using the 2^−ΔΔCt^ method [20], with assays conducted in triplicate with three technical replicates per sample. Target gene primers (Table 1) were obtained [19] from Integrated DNA Technologies (IDT, Coralville, IA, USA).

### 2.8. Statistical Analysis

For each sample, assays were performed in triplicate and reported as average values ± SE. To analyze the data, a one-way analysis of variance (ANOVA) was used and mean separation was performed via the Least Significant Difference (LSD) test at a 5% error rate using the Statgraphic v19.0 software.

## 3. Results and Discussion

### 3.1. Polyphenol Profiles from Aronia Berries Using LC-MS

**Figure 1:**
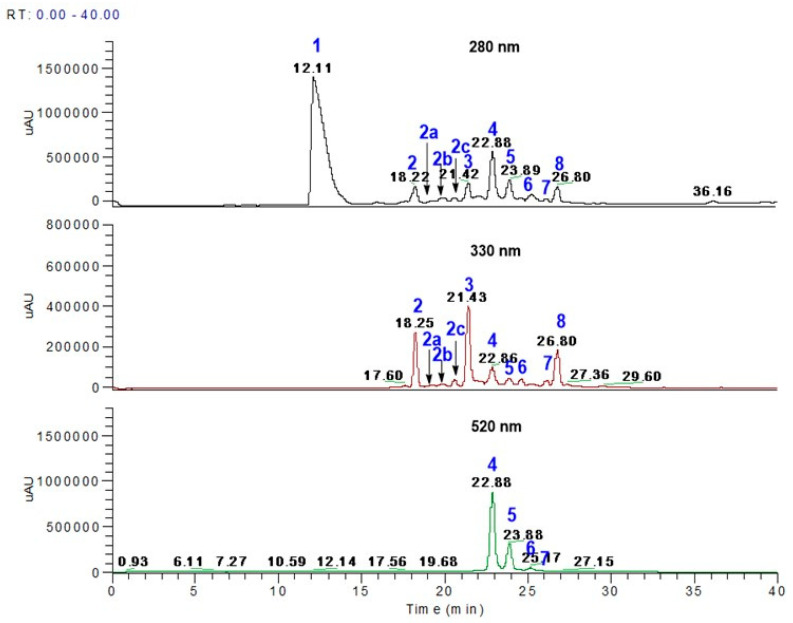
LC chromatograms of extracts of aronia polyphenol extracts. Individual peaks of phenolic compounds (peak numbers 1, 2, 2a–2c, and 3–8 in blue) were identified via LC-MS analysis and are reported in Table 2.

Herein, we tentatively identify the individual polyphenol compounds found in methanolic extracts of freeze-dried aronia samples via LC-MS analysis (Figure 1, Table 2); however, confirmation is encouraged through NMR analysis. Peak **1** (12.11 min) showed a deprotonated ion [M-H]^−^ at *m*/*z* 353 in the negative mode ionization and yielded a major fragment at *m*/*z* 191 [M-H-162]^−^ and *m*/*z* 179 [M-H-174]^−^; hence, the peak was identified as 5-*O*-caffeoyl quinic acid (5-CQA). Moreover, 5-CQA is reported to exhibit potent hydroperoxyl radical scavenging capacity in physiological media, both polar and lipidic, with rate constants higher than those of common antioxidants [26]. Peak **2** (18.22–18.25 min) also gave a deprotonated ion [M-H]^−^ at *m*/*z* 353 and gave fragments at *m*/*z* 191 [M-H-162]^−^ and *m*/*z* 179 [M-H-174]^−^; hence, the compound was identified as 3-*O*-caffeoyl quinic acid (3-CQA). It has been reported that 3-CQA has strong scavenging activity on superoxide anion radicals and an inhibitory effect against the oxidation of methyl linoleate [27], and shows higher antioxidant activity compared to 5-CQA [28].

Peak **2a** (19.20–19.22 min) gave [M-H]^−^ at *m*/*z* 625 and major fragments at [M-H-324]^−^ *m*/*z* 301 and [M-H-162]^−^ *m*/*z* 463; hence, the compound was identified as a quercetin dihexose. Peak **2b** (19.85–19.88 min) gave [M-H]^−^ at *m*/*z* 595 and yielded fragments at *m*/*z* 301 and *m*/*z* 463; hence, the compound was identified as quercetin-3-*O*-vicianoside. This compound has been reported to have wound-healing effects [29].

Peak **2c** (20.35–20.36 min) gave [M-H]^−^ at *m*/*z* 609 and yielded a fragment at *m*/*z* 301; hence, this compound was identified as quercetin-3-*O*rutinoside (rutin). Rutin is considered a potent antioxidant and to have anti-inflammatory properties [30]. Peak **3** (21.43 min) gave [M-H]^−^ at *m*/*z* 463 and fragments at *m*/*z* 287 and 421; hence, the compound was identified as Eriodictoyl gluconoride (EDG). EDG has been reported to have strong antiallergic properties, modulating gene and protein expressions in FcεRI-mediated human basophilic KU812F cells [31]. Peak **4** (22.88 min) gave [M-H]^−^ at *m*/*z* 447 and fragments at *m*/*z* 287 and at 211; hence, the compound was identified as cyaniding-3-*O*-galactoside (Cy3Gal). Cy3Gal is reported to have strong antioxidant and anti-inflammatory properties [32].

Peak **5** (23.88–23.89 min) gave [M-H]^−^ at 447 and yielded fragments at *m*/*z* 287 and 225; hence, the compound was identified as cyanidin-3-*O*-glucoside (C3G). C3G is known to have antioxidant and anti-inflammatory properties and has shown to protect the brain and improve cognitive function in a APPswe/PS1ΔE9 transgenic mice model [33]. Peak **6** (25.17 min) gave [M-H]^−^ at *m*/*z* 417 and yielded fragments at [M-H-130]^−^ *m*/*z* 287 and the fragment ion at *m*/*z* 225. Hence, compound 6 was identified as cyanidin-3-*O*-arabinoside (C3A). C3A has been shown to suppress DHT-induced dermal papilla cell senescence by modulating p38-dependent ER–mitochondria contacts through the prevention of ROS signaling caused by the NOX complex [34]. Peak **7** (26.18 min) gave [M-H]^−^ at *m*/*z* 417 and yielded a fragment at [M-H-130] *m*/*z* 287; hence, the compound was identified as cyanidin-3-*O*-xyloside.

Peak **8** (26.80 min) gave [M-H]^−^ at *m*/*z* 463 and fragments at [M-H-162]^−^ *m/z* 301 due to the loss of hexoside; hence, the compound was identified as quercetin hexoside.

The aronia phenolics identified in the present study and shown in Table 2 are similar to those reported previously [35,36,37,38,39]. The total phenolic content was the sum of individual phenolics present in the freeze-dried aronia berry (AroBerry^®^), giving ~7892 mg phenolics/100 g of freeze-dried sample (Table 2). In general, chlorogenic acids were the major phenolics present in the aronia berry, with 5-*O*-caffeoylquinic acid and 3-*O*-caffeoylquinic acid representing ~39.8 and 2.2%, respectively, while cyanidin derivatives showed major contributions overall, with cyanidin-3-*O*-galactoside, cyanidin-3-*O*-glucoside, cyanidin-3-*O*-arabinoside, and cyanidin-3-*O*-xyloside representing ~36.6, 10.6, 1.6, and 1.2%, respectively. Quercetin derivatives were present in smaller quantities with quercetin dihexoside, quercetin hexoside, rutin, and quercetin-3-*O*-vicianoside representing ~1.7, 2.1, 0.3, and 0.1%, respectively. Furthermore, the flavonoid Eriodictyol 7-*O*-β-glucuronide represented ~3.6%. (Table 2).

The phenolic content determined by in the Folin assay gave ~7622 mg phenolics/100 g of freeze-dried aronia berry (similar to the sum of individual phenolics determined by LCMS), while the ORAC assay gave values of ~64,544 μmol Trolox/100 g of freeze-dried aronia berry, which is within the range of antioxidant activity reported previously for aronia species [40]. The MeOH/acetone/H_2_O (5:4:1) aronia extraction gave a ~68.3% yield, and this aronia phenolic-rich extract was used for the cell assays. Overall, the phenolic content of the aronia extract determined by the Folin assay showed values of ~11.4 μg phenolics/100 μg of aronia extract.

### 3.2. Colon Cell Viability, Reactive Oxygen Species (ROS), and Effects in Pro-Inflammatory Genes

**Figure 2:**
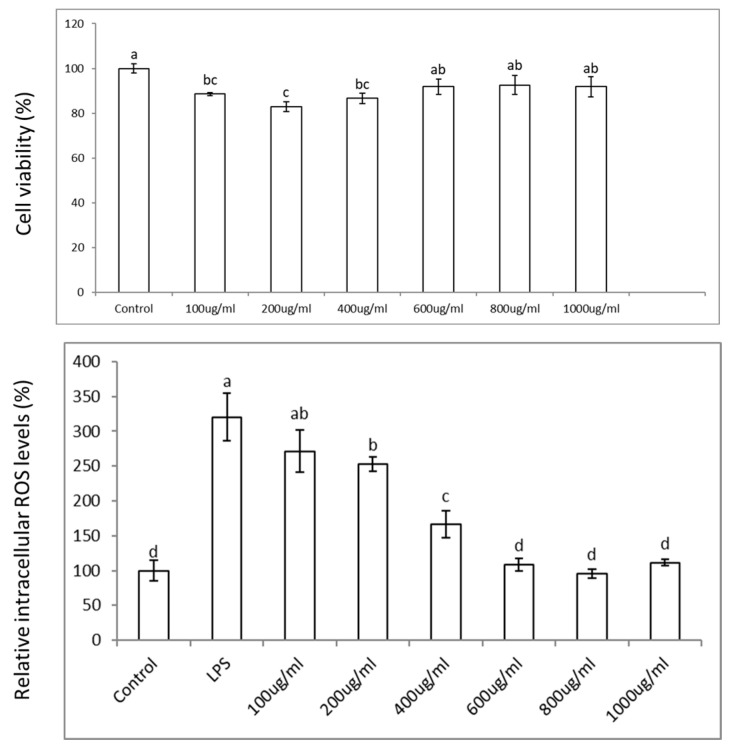
Colon epithelial cell responses to aronia berry extract treatment. Caco-2 cell viability (8 × 10^3^ cells/well) was evaluated following 24 h exposure to aronia extract (0–1000 µg/mL) using the MTS assay. For oxidative stress assessment, Caco-2 cells (8 × 10^3^ cells/well) were pretreated with aronia extract (0–1000 µg/mL) for 5 h prior to 19 h co-incubation with LPS (50 µg/mL). Statistical differences between treatments are indicated by different letters (*p* < 0.05, ANOVA with LSD post hoc test).

In this section, we studied the anti-inflammatory properties of freeze-dried aronia berries’ (AroBerry^®^) phenolic compounds in colon cells stimulated with an inflammatory bacterial lipopolysaccharide (LPS). The MTS test showed that phenolic extracts in the range of 100–1000 µg/mL (~11.4–114 µg phenolics/mL) did not affect cell viability in colon cells (Figure 2); thus, this range was used for the ROS and gene expression assays to determine their effects in preventing oxidative stress and inflammation, respectively, when colon cells were exposed to LPS.

**Figure 3:**
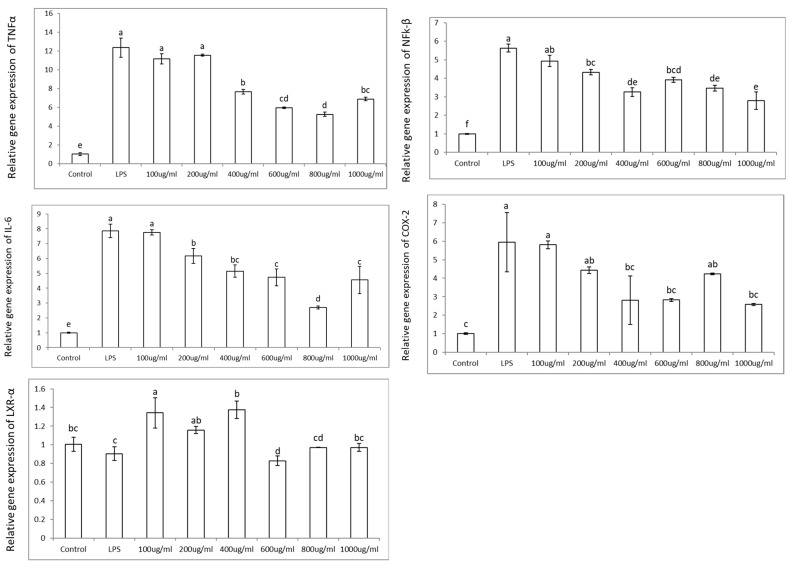
Anti-inflammatory effects of aronia extract in intestinal epithelial cells. Gene expression analysis of inflammatory mediators (*NFkβ*, *TNFα*, *IL-6*, and *COX-2*) and nuclear receptor *LXRα* was performed in Caco-2 cells (0.5 × 10^6^ cells/well) following sequential treatment with aronia extract (100–1000 µg/mL, 5 h pretreatment) and LPS (50 µg/mL, 19 h co-incubation). All extracts were prepared in growth medium containing 0.5% DMSO (vehicle control). Letters denote statistically significant differences between treatments (*p* < 0.05), as determined by ANOVA with LSD post hoc testing.

ROS increased in Caco-2 cells stimulated with LPS by ~225% and decreased in a dose-dependent manner when these cells were co-incubated with the aronia phenolic extracts in the range of 100–1000 µg/mL, showing a sharp decrease until ~600 µg/mL, with no further decrease thereafter. The effective reduction in ROS to levels similar to the control values at 600 µg/mL of aronia phenolic extract is equivalent to 68.4 µg phenolics/mL (Figure 2). These results confirm that aronia phenolic extracts reduce the oxidative stress induced by LPS and show potential to reduce inflammation. To further explore the latter, Caco-2 cells were exposed to LPS and showed an increase in the expression of pro-inflammatory genes *NFk-β*, *TNFα*, *IL-6*, and *COX2* by ~5.5, 12, 8, and 6 times, respectively. However, when colon cells were treated with aronia extracts at a range of 100–1000 µg/mL, there was an anti-inflammatory response in the expression of the pro-inflammatory genes *NFk-β*, *TNFα*, *IL-6*, and *COX2* (Figure 3) corresponding to the reduction in the ROS levels, shown earlier (Figure 3). These reductions were in the range of ~50% and in some cases similar to the control levels. For instance, the reduction in the expression of pro-inflammatory genes induced by aronia extracts varied according to the specific gene; thus, for *NFk-β*, *TNFα, IL-6*, and *COX2*, the reduction values were ~50, 50, 69, and 55%, respectively.

On the other hand, when colon cells were treated with LPS, there was a slight decrease in the genetic expression of nuclear receptors *LXRα* by ~10%. Furthermore, when LPS-challenged colon cells were treated with aronia extracts, the gene expression of *LXRα* levels increased by ~30% at aronia extract concentrations as low as 100 µg/mL and up to 400 µg/mL (Figure 3), suggesting that the activation of nuclear receptors is partially involved in the inactivation of NFkβ during the anti-inflammatory response in colon cells.

These results suggest that the anti-inflammatory mode of action of the aronia extract on colon cells is mainly through the reduction in oxidative stress, as well as by modulating nuclear receptors LXRα.

**Figure 4:**
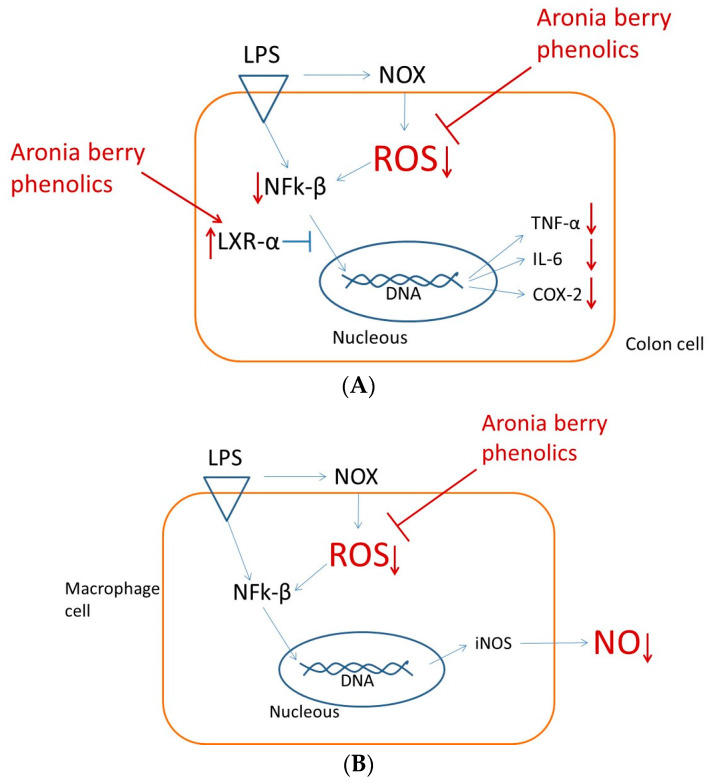
Aronia extract’s dual anti-inflammatory mechanisms in different cell types. Bacterial lipopolysaccharide (LPS) initiates inflammatory signaling by binding TLR4 receptors in both colonic and macrophage cells, activating NOX enzymes that generate ROS and subsequently trigger NFκB-mediated transcriptional activation of pro-inflammatory genes. (**A**) In colonic cells, aronia extract operates through complementary pathways: reducing ROS generation while simultaneously enhancing LXRα expression, with both actions converging to attenuate NFκB activation and thereby downregulate pro-inflammatory cytokines (TNF-α and IL-6) and enzyme COX-2. (**B**) In macrophages, aronia extract primarily functions by suppressing ROS production, which diminishes NFκB activation and consequently reduces the expression of iNOS, the enzyme responsible for NO synthesis. Up and downward arrows indicate up- and down-regulations, respectively.

Accordingly, bacterial lipopolysaccharide (LPS) initiates inflammatory signaling in colonic cells through TLR4 receptor binding, which activates NADPH oxidase and promotes ROS generation, subsequently triggering NFκB activation. Phosphorylated NFκB upregulates pro-inflammatory mediators, including TNF-α, IL-6, and COX-2. Aronia extract treatment significantly attenuates the ROS levels, potentially through direct radical scavenging through constituent polyphenols and/or the enhancement of cellular antioxidant defenses. This ROS reduction diminishes NFκB phosphorylation and the corresponding pro-inflammatory gene expression. Additionally, aronia polyphenols elevate LXRα expression, providing a secondary anti-inflammatory mechanism through NFκB inhibition. Thus, aronia extracts exhibit dual anti-inflammatory activity in colonic cells by modulating both oxidative stress and nuclear receptor signaling pathways (Figure 4A). The hypothetical model herein would need validation at the protein level to confirm the proposed mode of action.

### 3.3. Macrophage Cell Viability, Reactive Oxygen Species (ROS), and Nitric Oxide (NO) Production

**Figure 5:**
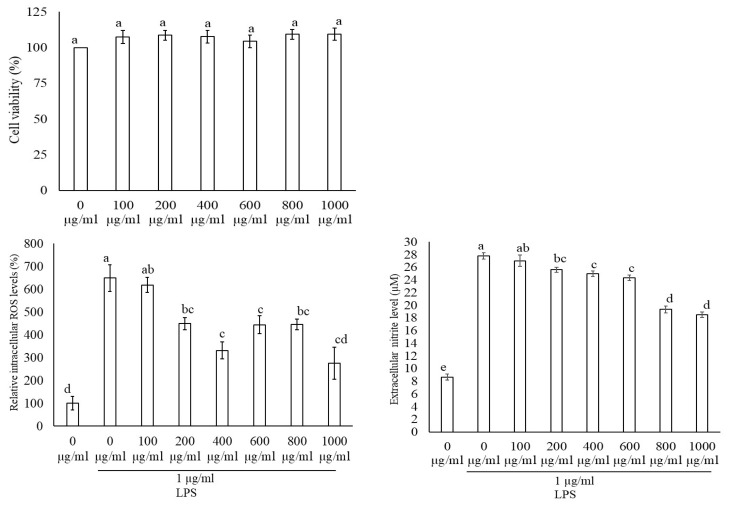
Macrophage cell responses to aronia extract exposure. Raw 264.7 macrophages (5 × 10^4^ cells/well) were assessed for viability after 24 h treatment with aronia extract (0–1000 µg/mL). For ROS and NO measurements, cells were pretreated with extract (0–1000 µg/mL) for 5 h followed by 19 h co-incubation with LPS (1 µg/mL). All extracts were prepared in growth medium containing 0.5% DMSO (vehicle control). Statistical significance (*p* ≤ 0.05) between treatments is indicated by different letters, determined via ANOVA with LSD post hoc analysis.

The anti-inflammatory properties of phenolic compounds from freeze-dried aronia berries (AroBerry^®^) were explored in macrophage cells stimulated with an inflammatory bacterial lipopolysaccharide (LPS). Initial studies using the MTS test showed that phenolic extracts in the range of 100–1000 µg/mL (~11.4–114 µg phenolics/mL) did not affect cell viability in macrophage cells (Figure 5). Accordingly, this range was used for the ROS and NO assays to determine their effects in preventing oxidative stress and inflammation, respectively, when macrophages were exposed to LPS.

Macrophage cells stimulated with LPS showed an increase in ROS by ~550%; however, ROS decreased in a dose-dependent manner when these cells were co-incubated with the aronia phenolic extracts in the range of 100–1000 µg/mL, showing a sharp decrease at ~200 µg/mL, with a smaller decrease thereafter. The effective reduction in ROS to levels similar to the control values was observed at 1000 µg/mL of aronia phenolic extract, which is equivalent to 114 µg phenolics/mL (Figure 3). Furthermore, for macrophage cells challenged with LPS, there was an increase in the NO levels by ~250%; however, these NO values decreased gradually when cells were treated with aronia phenolic extracts in the range of 100–1000 µg/mL in a dose-dependent manner. NO did not reach similar values to the controls, remaining ~137% higher at 1000 µg/mL (Figure 5). These results would suggest that aronia phenolic extracts reduce the oxidative stress induced by LPS and show the potential to reduce inflammation, as shown in the reduction in NO in macrophages (Figure 4B).

Overall, when microbiota dysbiosis takes place due to an altered diet, LPS is produced, inducing inflammation in the intestinal gut that may induce a leaky intestinal epithelium, allowing LPS to reach the blood circulatory system and induce inflammation in circulating macrophages [41]. This low-grade inflammation conduces a normal healthy state to a pre-disease state by creating an inflammatory lipotoxic milieu, which through a feedback loop mechanism, progresses to a chronic inflammation state [41]. The inflammatory lipotoxic milieu could potentially be decreased via an inflammation resolution mechanism, as observed in acute inflammation. In part, it is likely that the levels reached by the chronic inflammation state will eventually trigger different chronic diseases over time.

**Figure 6:**
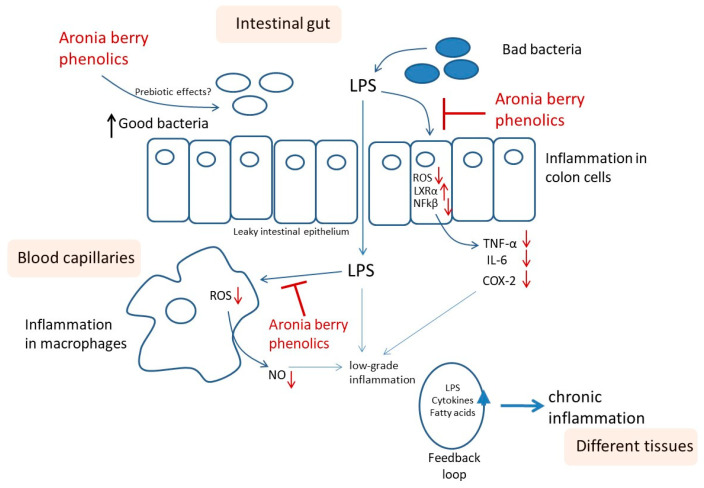
Proposed mechanistic framework for aronia phenolics’ multitargeted anti-inflammatory actions. Aronia berry constituents may prevent low-grade inflammation through three complementary pathways: (1) potential prebiotic activity (requiring confirmation); (2) attenuation of LPS-triggered inflammatory responses in intestinal epithelial cells following diet-induced dysbiosis; and (3) suppression of inflammatory cascades in macrophages exposed to circulating LPS that has traversed a compromised intestinal barrier. This latter mechanism may be particularly significant where circulating LPS activates peripheral macrophages, potentially initiating systemic low-grade inflammation that subsequently promotes chronic inflammatory processes across multiple tissues through a feedback loop mechanism. Up and downward arrows indicate up- and down-regulations, respectively.

In the present study, the LPS-induced pro-inflammatory cytokines TNFα and IL-6, enzyme COX-2 activity from colon cells, and NO from macrophages may contribute to the inflammatory lipotoxic milieu and the feedback loop (Figure 6). Thus, the importance of reducing these basal inflammatory levels with phenolics is observed in the present study.

A recent integrative model linking diet, medicine, and chronic disease progression describes how low-grade systemic inflammation transitions health status from normal to pre-disease, with chronic inflammation ultimately leading to disease manifestation [24]. This framework suggests that controlling inflammatory responses offers both preventive and therapeutic opportunities. Our findings demonstrate that aronia extract mediates cell type-specific anti-inflammatory mechanisms, with its bioactive constituents exhibiting multifaceted activities across different cellular targets. These properties position aronia extracts as potential dietary interventions for mitigating low-grade inflammation (Figure 6).

Further investigation is needed to identify which phenolic fractions in aronia berries possess the highest bioactivity, similar to studies on Clitoria ternatea, where specific flavonoid and anthocyanin fractions demonstrated anti-inflammatory effects [42]. Research on microbiota-derived metabolites from aronia extracts is also warranted, considering recent findings that urolithins (microbial metabolites of ellagic acid) reduce adipocyte lipid accumulation and attenuate LPS-induced inflammation [43]. Enriched phenolic extracts should be evaluated for potential digestive enzyme inhibition, while protein-level validation of the current gene expression findings would further elucidate aronia’s mechanisms of action. These findings will provide the foundation for subsequent in vivo studies using mouse models to confirm these anti-inflammatory properties, assess improvements in chronic conditions like metabolic syndrome, and establish appropriate dosages for future clinical trials [44,45].

## 4. Conclusions

In the present study, 11 phenolic compounds were identified in the chemical profile of freeze-dried aronia berries (AroBerry^®^) through LC-MS analysis. The extract demonstrated anti-inflammatory activity in LPS-challenged colon cells by suppressing oxidative stress and downregulating pro-inflammatory mediators (*NFkβ*, *TNFα, IL-6*, and *COX2*) while enhancing *LXRα* expression. In macrophages, the extract similarly reduced oxidative stress, resulting in decreased NO production. Notably, aronia extracts exhibited no cytotoxicity in either cell type across concentrations from 100 to 1000 μg/mL. The substantial phenolic content appears to be responsible for the observed multifaceted anti-inflammatory effects. These findings suggest potential applications of aronia berries in preventive dietary strategies against inflammation-associated chronic diseases, warranting pre-clinical studies to determine the appropriate dosage recommendations.

## Figures and Tables

**Table 1:** Primer sets for gene expression studies.

COX2	Fw-ACATCGATGTCATGGAACTG Rv-GGACACCCCTTCACATTATT
IL-6	Fw-TGACAACCACGGCCTTCCCT Rv-AGCCTCCGACTTGTGAAGTGGT
LXRα	Fw-AAGCCCTGCATGCCTACGT Rv-TGCAGACGCAGTGCAAACA
β-actin	Fw-CCCAGGCATTGCTGACAGG Rv-TGGAAGGTGGACAGTGAGGC
TNFα	Fw-ACTGGCAGAAGAGGCACTCC Rv-CGATCACCCCGAAGTTCA
NFKβ	Fw-GGTGGAGGCATGTTCGGTA Rv-TGACCCCTGCGTTGGATT

**Table 2:** Identification of phenolic compounds from freeze-dried aronia berries via LC-MS analysis.

Peak No.	Retention Time (min)	M-H	* MS Fragments	Aronia Berry Phenolics (mg/100 g)	Identification
1	12.11	353	**191**, 179	3114.8 ± 4	5-*O*-caffeoylquinic acid
2	18.22–18.25	353	**191**, 179	177.3 ± 2.4	3-*O*-caffeoylquinic acid
2a	19.20–19.22	625	**301**, 463	139.9 + 0.4	Quercetin dihexose
2b	19.85–19.88	595	**301**, 463	11.5 ± 0.3	Quercetin-3-*O*-vicianoside
2c	20.35–20.36	609	**301**	25.7 ± 0.0	Quercetin-3-*O*-rutinoside
3	21.43	463	**287**, 421	289.4 ± 0.9	Eriodictyol 7-*O*-β-glucuronide
4	22.88	447	**287**, 211	2890.3 ± 16.1	Cyanidin-3-*O*-galactoside
5	23.88–23.89	447	**287**, 225	839.6 ± 2.3	Cyanidin-3-*O*-glucoside
6	25.17	417	**287**, 225	129.9 ± 0.2	Cyanidin-3-*O*-arabinoside
7	26.18	417	287	100.4 ± 0.4	Cyanidin-3-*O*- xyloside
8	26.80	463	301	172.8 ± 0.7	Quercetin hexoside

* Note: Bold numbers indicate the base peaks in MS^n^ spectra.

## Data Availability

The original contributions presented in the study are included in the article, further inquiries can be directed to the corresponding author.

## Abbreviations

ROS	Reactive oxygen species
NO	Nitric oxide
LPS	Lipopolysaccharide
NFkβ	Nuclear factor kappa β
TNFα	Tumor necrosis factor alpha
IL-6	Interleukin 6
COX2	Cyclooxygenase 2
LXRα	Liver x receptor alpha
RNA	Ribonucleic acid
PCR	Polymerase chain reaction
DMSO	Dimethyl sulfoxide
NOX	NADPH oxidase (nicotinamide adenine dinucleotide phosphate oxidase)
iNOS	Inducible nitric oxide synthase
TLR4	Toll-like receptor 4
